# The Commission on Social Determinants of Health: Tackling the Social Roots of Health Inequities

**DOI:** 10.1371/journal.pmed.0030106

**Published:** 2006-05-23

**Authors:** Alec Irwin, Nicole Valentine, Chris Brown, Rene Loewenson, Orielle Solar, Hilary Brown, Theadora Koller, Jeanette Vega

## Abstract

The goal of the commission, launched in March 2005, is to strengthen health equity by catalysing policy and institutional change to address the social determinants of health.

## The Challenge

Throughout the world, people who are vulnerable and socially disadvantaged have less access to health resources, get sicker, and die earlier than people in more privileged social positions. Health equity gaps are growing today, despite unprecedented global wealth and technological progress [ 1, 2].

The greatest share of health problems is attributable to the social conditions in which people live and work, referred to as the social determinants of health (SDH) [ 3–5]. Good medical care is vital to the well-being of populations, but improved clinical care is not enough to meet today's major health challenges and overcome health inequities. Without action on social determinants, those countries in greatest need will neither meet the health-related Millennium Development Goals nor achieve global targets for reducing chronic diseases such as cardiovascular diseases, cancer, and diabetes [ 6].

Problems are especially urgent in developing countries where the burden of chronic illnesses is growing rapidly on top of the burden of unresolved infectious epidemics. In developing countries—including Brazil, China, India, and Pakistan—death rates from chronic diseases already far exceed the combined death rates from communicable diseases, maternal and perinatal conditions, and nutritional deficiencies [ 7]. Modifiable risk factors for chronic illness—such as poor diets, alcohol abuse, and smoking—are often seen as individual “lifestyle choices.” But such choices are conditioned by patterns of material deprivation and social exclusion. Health-compromising behaviors are disproportionately concentrated in socially disadvantaged groups, both in developed and in developing countries [ 8]. Effective policy to tackle health challenges must address the underlying social conditions that make people who are disadvantaged more vulnerable [ 9, 10]. [Fig pmed-0030106-g001]

**Figure pmed-0030106-g001:**
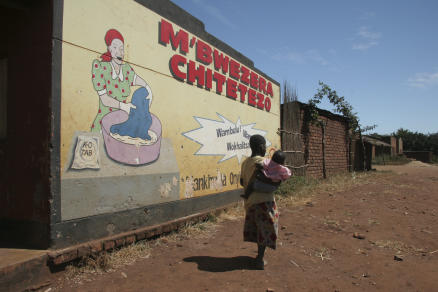
The social conditions in which children grow up shape their chances to be healthy (Image copyright: WHO. This photo may not be reproduced for commercial purposes; see http://www.who.int/about/copyright/en/index.html)

Ministries of health cannot transform social conditions single-handedly. However, the health sector can take leadership in advancing an approach to health policy that incorporates actions on SDH across government departments and the wider society. To advance this understanding of health as an integrative, cross-governmental goal and to support countries and global health partners in taking action on the social dimensions of health, the World Health Organization (WHO) has created the Commission on Social Determinants of Health (CSDH).

## How Will the Commission Operate?

The CSDH was formally launched in March 2005 and will operate until May 2008. Its goal is to strengthen health equity. It aims to do so by catalyzing policy and institutional change to address SDH within countries, among institutions working in global health, and within WHO itself [ 11, 12].

The components of the CSDH include the commissioners, partner countries, evidence-gathering knowledge networks, civil society organizations, and global institutions. The “commissioners” are 20 people chosen for being innovators in science, public health, policymaking, and action for social change ( http://www.who.int/social_determinants/en). The commissioners' primary responsibilities are advocacy and political leadership. “Knowledge networks” of scientists and practitioners will compile evidence on policies and interventions to overcome the social barriers to health, with a focus on low-income countries. Knowledge networks cover themes including early child development, employment conditions, globalization, determinants of women's health, the health system as a social determinant, urban settings, social exclusion, and measurement issues related to SDH policies. A dedicated knowledge network will look at how disease control programs on epidemics such as HIV/AIDS, tuberculosis, and malaria can integrate action on social determinants into their strategies.

Work at the national level is at the heart of the commission process: the CSDH will collaborate with countries in a variety of different ways. These include creating awareness and understanding of SDH among political leaders and stakeholders, capacity building and implementing specific interventions, and helping countries adopt comprehensive health and development policies oriented toward SDH. For best results, such policies require action across a range of sectors, such as finance, labor, education, and transportation, as well as health. While working with all interested countries, the CSDH is building intensive partnerships with one particular group of countries committed to comprehensive, cross-governmental action on SDH. During the commission's first year of activity, such partnerships were being formed with one to two countries in each of WHO's six global regions.

The political sustainability of social determinants policies at the national level depends on the support of civil society and communities. The CSDH has asked networks of civil society organizations to develop strategies for civil society collaboration with the commission in all global regions. These strategies will be shaped through consultative processes involving a broad spectrum of civil society and grassroots groups. They will help ensure that the CSDH agenda reflects communities' needs and knowledge, and that informed social demand supports the policy change process in countries.

## What Would Success Look Like?

The commission aims to lever policy change by turning public health knowledge into pragmatic global and national policy agendas [ 13]. Three years from now, we hope to have achieved the following outcomes: (1) SDH will be incorporated into national debates and policy processes in a growing number of countries, particularly in the developing world; (2) the opportunities for policy action on SDH, and the costs of not acting, will be widely known and discussed; (3) CSDH partner countries will be implementing policies on SDH and sharing results; (4) scientific knowledge on SDH will be consolidated, knowledge gaps clarified, and appropriate directions for ongoing research identified; (5) a WHO reference group linked to the commission will have presented detailed recommendations on how to incorporate SDH sustainably at WHO; and (6) SDH will inform WHO policy dialogue and technical work at a national level.

## Obstacles and Opportunities

Until now, systematic research on policies that work to reduce health inequities has been limited [ 14–16]. The CSDH faces significant challenges in attempting to fill this gap and to supply policymakers and program implementers with the evidence needed to choose, prioritize, sequence, and evaluate actions.

Even when effective policies are identified, they must still be “sold” politically. In an era when budgets for public-sector social programs are shrinking in many jurisdictions, proposals for a comprehensive effort to tackle SDH may face skepticism. Positioning health as a collective, cross-governmental concern demands effective strategies for multisectoral collaboration; historically, this has been difficult to achieve [ 17]. Moreover, even where political will to address SDH exists at a national level, national actors may find their options constrained by global forces. For example, national leaders' desire to raise public spending on social services relevant to SDH may clash with budget frameworks set by international financial institutions and donors. Likewise, a country's effort to enforce healthier working conditions in mines or factories may meet resistance from transnational corporations determined to minimize production costs [ 18, 19].

The current context offers opportunities for progress, however. Awareness of health disparities among social groups between and within countries is growing, along with the potential to mobilize social and political pressure for action [ 20]. The Millennium Development Goals have underscored the centrality of health on the development agenda and the need for multisectoral action to reach health objectives. Meanwhile, scientific understanding of the associations between social determinants and health inequities has progressed, and important examples of good practice in national health policy on social determinants have emerged [ 21, 22]. It will be important to document the policies and interventions on SDH that are proven to reduce health inequities. It will also be crucial to analyze the political processes that help to accelerate action on SDH.

This focus on collecting evidence, and on understanding political processes, implies that the CSDH will not simply produce a report and expect countries to adopt its recommendations because they are scientifically sound. Rather, policy recommendations are being shaped and “reality tested” through political collaboration with partner countries over the lifetime of the commission. The CSDH focuses its activities on specific social determinants that deepen health inequities, and for which (1) effective policies and interventions exist, (2) interventions are politically and economically feasible, and (3) sufficient social and political support can be mobilized to give the issues priority on national policy agendas. The commission will give greatest emphasis to policies that address major structural determinants of health, such as labor market policies and the education system, rather than “downstream” actions more focused on individual risk factors. Key action areas are shown in Box 1. Examples of how the CSDH is working with two countries—Chile and Kenya—are shown in Box 2.

## Moving Forward

The CSDH was launched in March 2005 in Chile and has held subsequent meetings in Egypt, India, and Iran. Commissioners will next meet in June 2006, in Kenya. Currently, knowledge networks have begun their operations, and joint work plans are being developed with a lead group of partner countries. Brazil, for example, has launched its own national commission on social determinants, which will work closely with the global CSDH to stimulate uptake of SDH policies nationally, across the Americas, and in the Portuguese-speaking countries of Africa. A WHO reference group appointed by the director-general has begun to develop sustainable strategies to incorporate SDH perspectives into WHO policies and programs.

When reduced to their most fundamental formulation, scientific claims on social determinants often seem to be common sense. One hardly needs sophisticated epidemiological models to see that it is inefficient to treat children for diarrhea or respiratory ailments, and then send them back to slum-like living conditions that virtually guarantee new bouts of infection. But translating such common sense into public policy has proven difficult in many settings. The CSDH seeks to understand the reasons for this failure and to learn from cases where action on SDH has worked.

**Box 1. Five Key Action Areas for the CSDH**

- Improving living and learning conditions in early childhood
- Strengthening social programs to provide fairer employment conditions and access to labor markets, particularly for vulnerable social groups
- Policies and interventions to protect people in informal employment—that is, those who work without formal contracts or social protections, often in sectors outside government regulation, such as subsistence farming, household-based enterprises, and street vending
- Policies across sectors to improve living conditions in urban slums
- Programs to address key determinants of women's health, such as access to education and economic opportunities

**Box 2. Examples of How the CSDH Is Working with Partner Countries**

**Chile**

- Strengthening the focus on equity in national health goal setting and monitoring
- Developing equity-oriented approaches in occupational health, with special attention on sectors of economic activity where formal contracts are often not used and that may fall outside government regulation (e.g., seasonal agricultural work)
- Incorporating SDH analysis into a planned national multisectoral strategy for the social protection of children

**Kenya**

- Developing and testing governance mechanisms to coordinate cross-sectoral work on SDH
- Capacity building within the Ministry of Health for effective leadership in cross-sectoral SDH policy
- Creating mechanisms for sustainable government investment in the social determinants by incorporating SDH and health equity perspectives into the National Medium-Term Expenditure Framework, a government planning tool that helps guide policy priorities and spending choices over a period of several years

## Abbreviations

CSDH: Commission on Social Determinants of Health
SDH: social determinants of health
WHO: World Health Organization
